# Mammary tissue proteomics in a pig model indicates that dietary valine supplementation increases milk fat content via increased de novo synthesis of fatty acid

**DOI:** 10.1002/fsn3.2574

**Published:** 2021-09-16

**Authors:** Long Che, Mengmeng Xu, Kaiguo Gao, Li Wang, Xuefen Yang, Xiaolu Wen, Hao Xiao, Mengyun Li, Zongyong Jiang

**Affiliations:** ^1^ College of Animal Science and Technology Henan University of Animal Husbandry and Economy Zhengzhou China; ^2^ State Key Laboratory of Livestock and Poultry Breeding Key Laboratory of Animal Nutrition and Feed Science in South China Ministry of Agriculture, Guangdong Public Laboratory of Animal Breeding and Nutrition Guangdong Key Laboratory of Animal Breeding and Nutrition Institute of Animal Science Guangdong Academy of Agricultural Sciences Guangzhou China

**Keywords:** gilt, milk fat, proteomics, valine

## Abstract

Milk fat is a major source of energy that determines the growth of neonates. Recently, studies have shown that valine is closely related to lipid metabolism. Therefore, this study was designed to investigate the effects of dietary valine supplementation on milk fat synthesis using a pig model. Thirty gilts were allotted to low (LV, total valine:lysine = 0.63:1), medium (MV, total valine:lysine = 0.73:1), and high (HV, total valine:lysine = 0.93:1) valine feeding levels from Day 75 of gestation till farrowing. The results demonstrated that the concentration of milk fat at Days 1, 3, and 7 of lactation in the HV group was higher than that in the MV and LV groups. The HV group had an increased (p < .05) proportion of total saturated and monounsaturated fatty acids than the other groups. Examination of mammary tissue proteomics in the HV and LV groups revealed 121 differentially expressed proteins (68 upregulated and 53 downregulated in the HV group). The upregulated proteins in the HV group were relevant to some crucial pathways related to milk fat synthesis, including fatty acid biosynthesis and metabolism, the AMPK signaling pathway, and oxidative phosphorylation. Furthermore, the key proteins involved in fatty acid synthesis (ACACA and FASN) were identified, and their expression levels were verified (p < .05) using Western blotting. Our findings revealed that dietary valine supplementation improves milk fat synthesis by modulating the expression of fatty acid synthesis–related proteins in mammary tissues.

## Graphical abstract

Dietary valine supplementation increases the content of milk fat in the colostrum Dietary valine supplementation increases the proportion of fatty acids in colostrum. Valine can activate the signaling pathway related to fatty acids synthesis. Valine promote fatty acids synthesis may via the upregulation of proteins ACACA and FASN

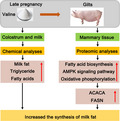

## 1 INTRODUCTION

Branched chain amino acids (BCAAs; valine, leucine, and isoleucine) are not only essential and limiting dietary amino acids that are building blocks for tissue proteins but also important signaling molecules that regulate physiological metabolism (Wu, 2013). Recent studies in humans and rats have highlighted that there is a significant correlation between the concentration of BCAAs in plasma and lipid metabolism homeostasis (Duan et al., 2018; Kainulainen et al., 2013; White et al., 2018). These studies suggest that BCAAs play a considerable role in fatty acid (FA) synthesis in adipose tissues. In animal and human nutrition, milk fat is a major factor determining the value of milk, which consists of more than 95% triglycerides, and is closely related to neonate growth and development (Innis, 2011). The mammary glands synthesize and secrete milk fat. The data from rats suggested that the rate of FA synthesis in adipose tissue was 345 μmol/h during nonlactation; however, it dropped sharply to 39 μmol/h during peak lactation. In contrast, the rate of FA synthesis in the mammary gland may be as high as 2950 μmol/h during peak lactation (Agius et al., 1979; Kanto & Clawson, 1980). Therefore, lactating mammary glands extend beyond adipose tissue as the most active lipid‐synthesizing and secreting organs in the body (Russell et al., 2007; Schwertfeger et al., 2003). Most studies indicate that the concentration of milk fat is mainly regulated by dietary FA supplementation. For example, increasing supplementation of lipids (Rosero et al., 2015), triglycerides (Azain, 1993), or conjugated linoleic acid (Krogh et al., 2012) in a lactation diet improved milk fat secretion. Currently, there are few reports on the role of BCAAs in regulating sow milk fat synthesis. Of all amino acids, uptake of BCAAs by sow mammary glands is considerably higher than the output in milk (uptake:output >1) (Manjarin, et al., 2012, indicating that BCAAs are extensively catabolized in lactating mammary tissues. Notably, the absorption of valine in the mammary gland is the highest among all amino acids during lactation, suggesting that valine plays a crucial role in mammary gland metabolism (Manjarin, Zamora, et al., 2012; Wang et al., 2019). Recently, research by our group found that valine enhanced lipid droplet synthesis in porcine mammary epithelial cells (Che, Xu, Gao, Zhu, et al., 2019; however, the effects and mechanism of dietary valine regulating milk fat synthesis in gilts remain unelucidated and need to be further investigated.

Therefore, this study explored the roles and molecular basis of valine in FA synthesis in the mammary glands of gilts. Using a pig model, we investigated the related factors using mammary tissue proteome analysis with data‐independent acquisition (DIA). Our results will offer new evidence for a better understanding of the beneficial effects and molecular basis of the regulation of milk fat synthesis in lactating animals.

## 2 MATERIALS AND METHODS

### 2.1 Animals and diets

Thirty primiparous gilts (Yorkshire ×Landrace, average weight 149.88 ± 4.28 kg, average age 248 ± 4.36 d) were used in this experiment. All gilts were synchronized for estrus and artificially inseminated according to a previous study (Che et al., 2020). During early (from Days 0 to 30) and middle (from Days 31 to 75) stages of gestation, gilts were fed 2.08 kg of a commercial diet (3.09 Mcal/kg digestible energy, 12.95% crude protein) per day. According to back fat thickness and body weight, gilts were randomly assigned to three dietary treatment groups on Day 75 of gestation.

Gilts in the three groups were fed corn–soy basal diet (LV group, 0.85% total lysine, 0.54% total valine, total valine:lysine = 0.63:1), MV group diet (0.85% total lysine, 0.62% total valine, total valine:lysine = 0.73:1), and HV group diet (0.85% total lysine, 0.79% total valine, total valine:lysine = 0.93:1), respectively. The MV group diet was formulated according to the valine requirement of gestation gilts recommended by the National Research Council (NRC, 2012). Valine was supplemented in MV and HV groups, with 98% purity of crystalline l‐valine (CJ Shenyang Biotech Co., Ltd., Liaoning, China). The experimental basal diet included 3.12 Mcal/kg DE and 13.60% CP from Day 75 of gestation to farrowing. All gilts were fed twice daily (08:00 and 17:00 h), and the daily feed was 2.32 kg/d from Days 75 to 90 of gestation and 2.72 kg/d from Day 91 to farrowing. The daily intake of valine in LV, MV, and HV was 12.53 g, 14.38 g, and 18.33 g from Days 75 to 90 of pregnancy and 14.69 g, 16.86 g, and 21.49 g from Day 91 to farrowing, respectively. Within 24 h of farrowing, the litter size was standardized to 10 piglets per gilt within the same treatment, depending on their availability and body weight.

### 2.2 Sample collection

Colostrum samples (30 ml) were collected within 2 h of the first piglet, as previously described (Che, Xu, Gao, Wang, et al., 2019. Milk samples (30 ml) were collected from all functional glands on Days 3, 5, 7, 10, 14, 17, and 21 of lactation. Milk samples were collected, as described in a previous study (Che, Xu, Gao, Wang, et al., 2019. The colostrum and milk samples were stored at −20℃ immediately till they were used for subsequent analysis. On Day 1 of lactation, 4 gilts from each group were anesthetized and mammary tissues were collected from the third mammary gland on the left side of the body, as previously described (Kirkwood et al., 2007).

### 2.3 Biochemical analyses

The concentration of fat in the colostrum and milk was measured using an ultrasonic milk analyzer (Milkyway‐CP2, Beijing, China). FA composition was determined using gas chromatography, as described earlier (Mossoba et al., 2001). The procedure involves lipid extraction, FA methylation, and gas chromatography. The FA content was determined by calculating the chromatographic peak area. The expression levels of the proteins were determined using the following steps. First, total proteins were extracted with RIPA buffer (Beyotime, Beijing, China) by centrifugation. Protein concentration in the supernatant fluid was measured using a BCA protein assay kit (Thermo Fisher Scientific, Waltham, MA, USA) following the manufacturer's instructions. Proteins (50 μg/lane) were separated using sodium dodecyl sulfate–polyacrylamide gel electrophoresis (SDS–PAGE) and transferred to polyvinylidene difluoride membranes. The membranes were blocked using QuickBlockTM Western buffer (Beyotime, Shanghai, China) at room temperature for 1 h, incubated with the primary antibodies at 4℃ overnight, and then incubated with the secondary antibody for 1 h at room temperature. The primary antibodies included anti‐acetyl‐CoA carboxylase (ACACA) (#3662s) antibody obtained from Cell Signaling Technology (Beverly, MA, USA), anti–fatty acid synthase (FASN) (ab128870) antibody purchased from Abcam (Cambridge, UK), and anti‐β‐actin (anm40032) antibody obtained from Amyjet Scientific (Abbkine, Wuhan, China).

### 2.4 Mammary tissue sample preparation

The mammary tissue samples from the LV and HV groups were ground into powder under liquid nitrogen and extracted using lysis buffer (100 mmol/L NH_4_HCO_3_ (pH = 8), 6 mol/L urea, and 0.2% SDS), followed by 5 min of ultrasonication on ice. The lysate was centrifuged at 12,000 × *g* for 15 min at 4℃. The supernatant was transferred to a clean tube, 10 mmol/L dithiothreitol was added to the samples and kept for 1 h at 56℃, and the solution was subsequently alkylated using iodoacetamide for 1 h at room temperature in the dark. Then, four times the volume of precooled acetone was added, and the samples were then vortexed and incubated at −20℃ for at least 2 h. The samples were centrifuged, and the precipitates were collected. After washing twice with cold acetone, the pellets were dissolved in dissolution buffer containing 0.1 mol/L triethylammonium bicarbonate (TEAB, pH = 8.5) and 6 mol/L urea. Protein concentration was determined using the Bradford protein assay kit (Bio‐Rad, CA, USA).

### 2.5 Peptide preparation and high‐performance liquid chromatography fractionation

The supernatant from each sample, containing precisely 0.12 mg of protein, was digested with Trypsin Gold (Madison, WI, USA) at 1:50 enzyme‐to‐substrate ratio. After 16 h of digestion at 37℃, the mixture sample (mixed sample) and the remaining peptides (single samples) were desalted with a C18 cartridge to remove urea, and then the desalted samples were dried using vacuum centrifugation. The mixed sample was fractionated using a C18 column (Thermo Fisher Scientific) on a Rigol L‐3000 HPLC system (Rigol, Beijing, China) operated at 1 ml/min, and the column oven was set at 50℃. Mobile phases A (2% acetonitrile, pH 10.0) and B (98% acetonitrile, pH 10.0) were used to develop a gradient elution. The eluates were monitored at 214 nm, collected in 1 tube per minute, and finally merged into 4 fractions. All fractions were dried under vacuum and reconstituted in 0.1% (v/v) formic acid in water. We added 0.2 µl of standard peptides to the fraction sample for subsequent analyses.

### 2.6 LC‐MS/MS analysis in the data‐dependent acquisition (DDA) mode

For transition library construction, shotgun proteomics analysis was performed using an EASY‐nLCTM 1200 UHPLC system (Thermo Fisher Scientific) coupled with an Orbitrap Q Exactive HF‐X mass spectrometer (Thermo Fisher Scientific) operating in the DDA mode. A sample volume containing 2 µg of total peptides from the fraction sample was reconstituted in 0.1% formic acid and injected into a homemade C18 Nano‐Trap column (2 cm × 100 µm, 3 µm). Peptides were separated using a homemade analytical column (15 cm × 150 µm, 1.9 µm) with a 120‐min linear gradient from 5% to 100% of eluent B (0.1% formic acid in 80% acetonitrile) in eluent A (0.1% formic acid in H_2_O) at a flow rate of 600 nl/min. The Q Exactive HF‐X mass spectrometer was operated in a positive polarity mode, with a spray voltage of 2.3 kV and capillary temperature of 320℃. Full MS scans ranging from 350 to 1500 m/z were acquired at a resolution of 60,000 (at 200 m/z), with an automatic gain control (AGC) target value of 3 × 10^6^ and a maximum ion injection time of 20 ms. The 40 most abundant precursor ions from the full MS scan were selected for fragmentation using higher energy collisional dissociation fragment analysis at a resolution of 15,000 (at 200 m/z), with an AGC target value of 5 × 10^4^, a maximum ion injection time of 45 ms, a normalized collision energy of 27%, an intensity threshold of 2.2 × 10^4^, and a dynamic exclusion parameter of 40 s.

### 2.7 LC‐MS/MS analysis in the DIA mode

The single sample was reconstituted in 0.1% formic acid, mixed with 0.2 µl standard peptides (Biognosys, MA, USA), and injected into the EASY‐nLCTM 1200 UHPLC system (Thermo Fisher) coupled with an Orbitrap Q Exactive HF‐X mass spectrometer (Thermo Fisher) operating in the DIA mode. The liquid conditions were the same as those described earlier. For DIA acquisition, MS1 resolution was set to 60,000 (at 200 m/z), whereas MS2 resolution was set to 30,000 (at 200 m/z). The m/z range was 350–1500 m/z, with 30 variable cycles. The full scan AGC target was set at 3 × 10^6,^ and the injection time was 50 ms. The DIA settings were NCE 27% and target value 1 × 10^6^, and the maximum injection time was set as auto to allow the mass spectrometer to always operate in the parallel ion filling and detection mode.

### 2.8 Proteomic data analysis

Analysis and visualization of DDA and DIA data were performed using the Proteome Discoverer 2.2 platform (PD 2.2; Thermo Fisher Scientific), Spectronaut version 9.0 (Biognosys, MA, USA), and the R statistical framework. DDA MS raw files were analyzed using PD 2.2, and peak lists were searched against the protein database. The false discovery rate was set to 5% for both proteins and peptides and was determined by searching a reverse database. MS1‐based label‐free quantification (LFQ) was performed using the maxLFQ algorithm (Cox et al., 2014). MS2‐based LFQ was performed by analyzing raw DIA data using Spectronaut. Data analysis was performed, as previously described (Bruderer et al., 2015).

### 2.9 Statistical analyses

Data were presented as mean ± *SEM*. Statistical analyses were performed using SPSS software (version 19.0; SPSS Inc., Chicago, IL, USA). For statistical comparisons among study groups, the milk fat concentration, FA composition, and protein expression from Western blotting were analyzed using ANOVA, and the Tukey test was used to determine the differences among the groups. The gilt was used as the experimental unit. For metabolomics analysis, fold change (FC) was calculated for each comparison studied, and a minimum value of 2.0, and values of p < .05 were established to consider the protein abundance as significantly different.

## 3 RESULTS

### 3.1 Effects of dietary valine supplementation on the concentration of milk fat

**FIGURE 1:**
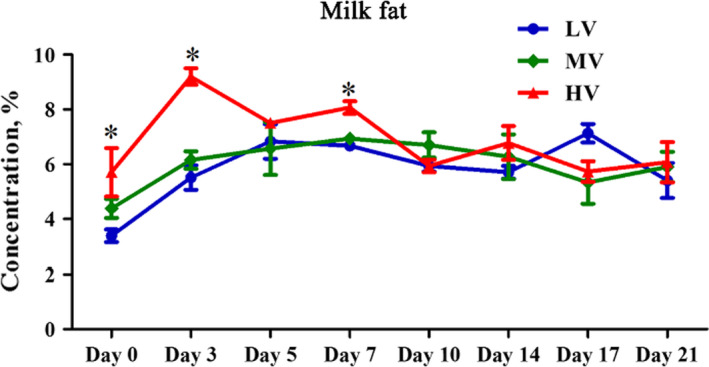
Effects of dietary valine supplementation on concentration of milk fat. Note: LV: total valine:lysine = 0.63:1; MV: total valine:lysine = 0.73:1; HV: total valine:lysine = 0.93:1. All data with error bars represent the mean ± *SEM*. * p < .05

Compared with gilts fed with the LV diet, those fed with the HV diet had an increased fat content (p < .05; increased by 68%) in the colostrum (Figure 1). Supplementation with HV diet markedly increased the fat content (p < .05) compared with the LV and MV groups on Days 3 and 7 of lactation; however, there were no differences among treatments (p > .05) from Days 10 to 21 (Figure 1).

### 3.2 | Effects of dietary valine supplementation on the FA composition of milk

For colostrum, higher proportions of C14:0, C18:3(n‐3), C18:3(n‐6), and total saturated FAs were observed in the HV group than those in the LV and MV groups (p < .05; Table 1). The proportion of C18:1 (n‐9) and total monounsaturated FAs was also higher in the HV group than in the LV group (p < .05; Table 1). However, there was no significant difference between treatments regarding the proportion of saturated and unsaturated FAs (p > .05) in the milk on Day 10 of lactation (Table 2).

**TABLE 1:** Effects of valine supplementation in diets on fatty acids composition of colostrum

Item	LV	MV	HV	p value
Saturated fatty acids				
C14:0	1.38 ± 0.06^b^	1.33 ± 0.08^b^	1.65 ± 0.07^a^	.021
C16:0	21.19 ± 0.21	22.11 ± 0.57	23.34 ± 0.18	.076
C17:0	0.36 ± 0.03	0.28 ± 0.05	0.31 ± 0.06	.546
C18:0	6.04 ± 0.43	5.58 ± 0.22	6.27 ± 0.26	.338
Total	29.98 ± 0.29^b^	29.30 ± 0.62^b^	31.57 ± 0.38^a^	.021
Monounsaturated fatty acids				
C16:1	2.12 ± 0.12	2.09 ± 0.04	2.12 ± 0.04	.950
C18:1(*n*−9)	32.01 ± 0.22^b^	33.43 ± 1.26^ab^	36.51 ± 1.25 ^a^	.035
Total	34.13 ± 0.21^b^	35.52 ± 1.23^ab^	38.63 ± 1.26 ^a^	.034
Polyunsaturated fatty acids				
C18:2(*n*−6)	25.88 ± 1.11	28.79 ± 1.56	29.53 ± 0.44	.106
C18:3(*n*−3)	1.00 ± 0.03 ^b^	1.11 ± 0.03 ^b^	1.24 ± 0.04 ^a^	.003
C18:3(*n*−6)	0.26 ± 0.02 ^b^	0.24 ± 0.01 ^b^	0.31 ± 0.01 ^a^	.007
C20:3(*n*−3)	0.31 ± 0.01	0.28 ± 0.01	0.28 ± 0.07	.490
C20:4(*n*−6)	1.10 ± 0.08	1.22 ± 0.05	1.25 ± 0.08	.388
Total	28.56 ± 1.11	31.62 ± 1.60	32.61 ± 0.52	.085

Note

Results were presented as mean values with their standard errors, *n* = 4. LV: total valine:lysine = 0.63:1; MV: total valine:lysine = 0.73:1; HV: total valine:lysine = 0.93:1.

**TABLE 2:** Effects of valine supplementation in diets on fatty acids composition of milk on Day 10 of lactation

Item	LV	MV	HV	p value
Saturated fatty acids				
C14:0	3.39 ± 0.07	3.32 ± 0.26	3.26 ± 0.20	.900
C16:0	30.79 ± 0.41	30.21 ± 1.15	28.49 ± 1.53	.387
C17:0	0.12 ± 0.01	0.13 ± 0.01	0.12 ± 0.01	.757
C18:0	3.76 ± 0.34	4.57 ± 0.59	4.78 ± 0.37	.281
Total	37.96 ± 0.75	38.24 ± 0.93	36.65 ± 1.39	.55
Monounsaturated fatty acids				
C16:1	7.28 ± 0.76	7.30 ± 0.80	7.67 ± 0.60	.913
C18:1(*n*−9)	29.34 ± 0.95	31.32 ± 2.41	34.68 ± 3.19	.320
Total	36.62 ± 1.02	38.62 ± 1.67	42.36 ± 3.35	.238
Polyunsaturated fatty acids				
C18:2(*n*−6)	20.46 ± 1.28	19.58 ± 1.18	18.45 ± 1.77	.625
C18:3(*n*−3)	1.25 ± 0.02	1.29 ± 0.04	1.26 ± 0.03	.625
C20:4(*n*−6)	0.45 ± 0.02	0.43 ± 0.04	0.47 ± 0.02	.722
C20:5(*n*−3)	0.30 ± 0.09	0.28 ± 0.09	0.27 ± 0.08	.976
C22:6(*n*−3)	0.50 ± 0.12	0.44 ± 0.06	0.36 ± 0.15	.628
Total	22.95 ± 1.16	22.02 ± 1.13	20.81 ± 1.93	.596

Note

Results are presented as mean values with their standard errors, *n* = 4. LV: total valine:lysine = 0.63:1; MV: total valine:lysine = 0.73:1; HV: total valine:lysine = 0.93:1.

### 3.3 Differentially expressed proteins in mammary tissue between the LV and HV groups on Day 1 of lactation

**FIGURE 2:**
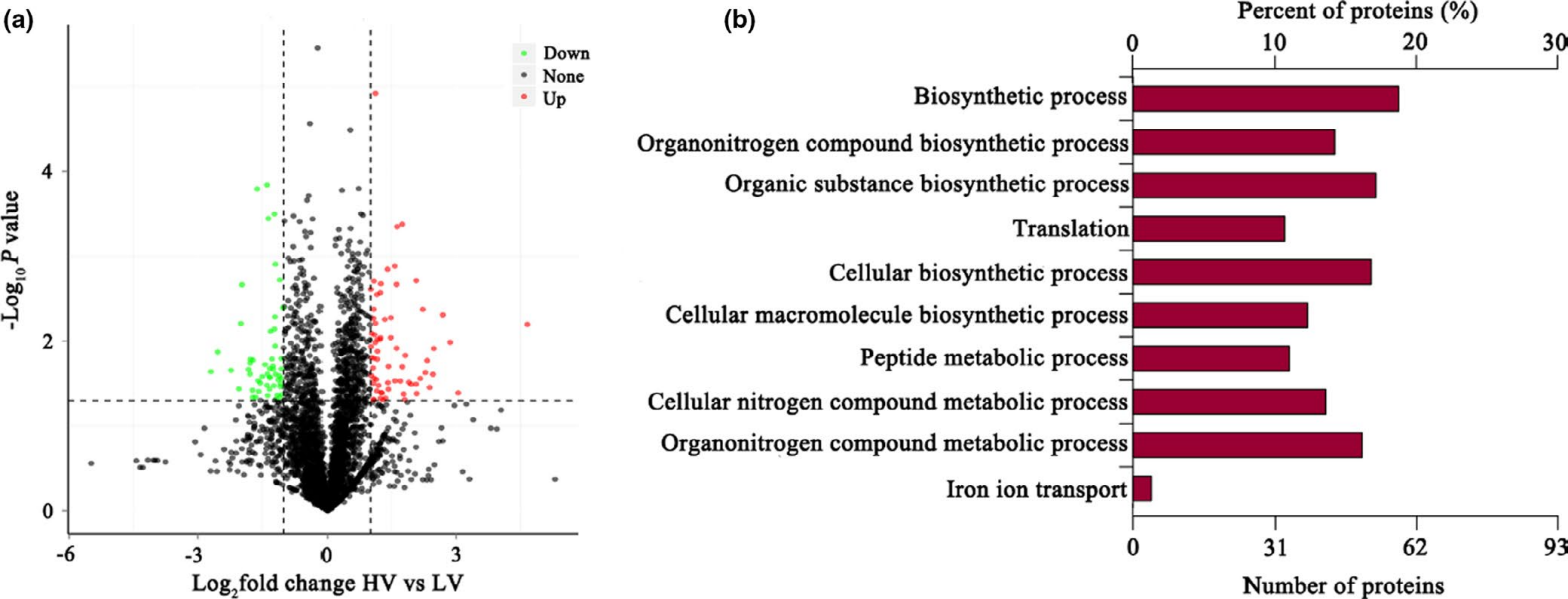
Significantly upregulated biological processes observed in mammary tissue of the HV group. Note: LV: total valine:lysine = 0.63:1; HV: total valine:lysine = 0.93:1. A: Principal component analysis (PCA) comparing the proteome of mammary tissues from the HV and LV groups, proteins with higher expression in the HV group are shown in red, lower in green, and no difference in black. B: the significantly enriched terms for the biological process

**FIGURE 3:**
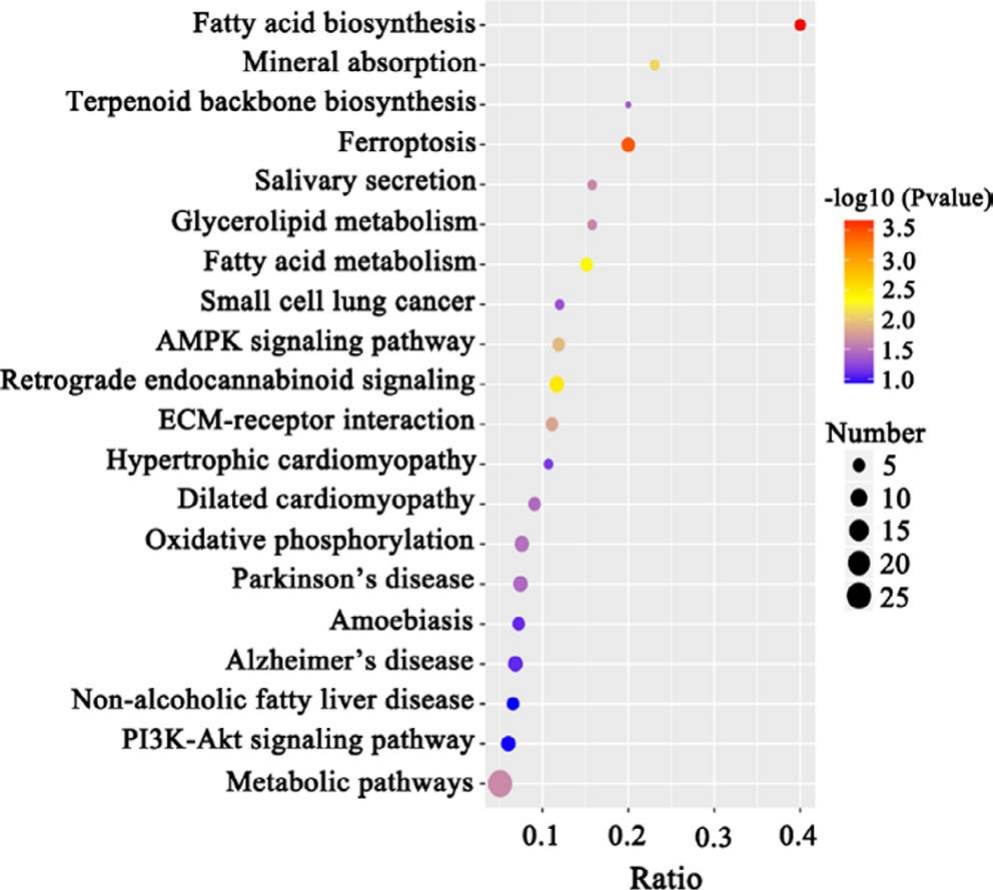
The top 20 upregulated enriched KEGG pathways observed in mammary tissue of the HV group. Note: LV: total valine:lysine = 0.63:1; HV: total valine:lysine = 0.93:1. Ratio: the ratio of the number of differential proteins to the number of total identified proteins under the corresponding pathway; number: the number of the differential proteins under the corresponding pathway

In total, 42,315 peptides were identified. Our approach resulted in the identification and quantification of 3,753 proteins (Table S1). Among the identified proteins, a total of 121 proteins showed a > 2‐FC between the LV and the HV groups (p < .05), of which 68 were upregulated and 53 were downregulated in the HV group when compared with the LV group (Table S2, Figure 2a). According to the results of gene ontology analysis, a total of 96 biological processes, 77 molecular functions, and 15 cellular components were enriched (FDR <0.1; Table S3). The most significantly enriched terms for biological processes included biosynthetic processes, organonitrogen compound biosynthetic processes, and organic substance biosynthetic processes (Figure 2b). With respect to the molecular functions and cellular components, we identified proteins mainly related to the regulation of enzyme activity, structural molecules activity, and ribosomes (Table S3). Among the differentially expressed proteins, 29 upregulated proteins in the HV group were categorized according to the clusters of orthologous groups (COG) function classification and were found to be primarily involved in carbohydrate metabolism, amino acid metabolism, lipid transport and metabolism, nucleotide transport and metabolism, inorganic ion transport and metabolism, energy production and conversion, and posttranslational modifications (Table 3). Among the upregulated proteins in the HV group, there were 9 proteins involved in lipid transport and metabolism, including FASN, ACACA, acylglycerol kinase (AGK), 3‐hydroxy‐3‐methylglutaryl coenzyme A synthase (HMGS), acyl‐CoA desaturase, and diphosphomevalonate decarboxylase; however, only 1 protein, asparagine synthetase, was involved in amino acid metabolism. By contrast, 26 proteins downregulated in the HV group, categorized according to the COG function classification, were found to be primarily involved in inorganic ion transport and metabolism, coenzyme transport and metabolism, transcription, posttranslational modifications, cell growth, and cell motility (Table 3). Among these downregulated proteins, there were 6 proteins involved in cell growth and cell motility. These data were then analyzed in the context of known genes obtained from the Kyoto Encyclopedia of Genes and Genomes (KEGG) database. The results showed that FA biosynthesis, FA metabolism, glycerolipid metabolism, the AMP‐activated protein kinase (AMPK) signaling pathway, oxidative phosphorylation, and the PI3K–Akt signaling pathway were significantly enriched and that these pathways were closely related to FA synthesis in mammary tissues (Figure 3). Interestingly, among these pathways, the proteins FASN and ACACA were enriched and involved in FA biosynthesis, FA metabolism, and the AMPK signaling pathway.

**TABLE 3:** Differentially expressed proteins between HV and LV groups in the mammary tissue on Day 1 of lactation

Accession no	Protein name	Gene symbol	Fold change	p value	Categories
I3LR64	Uncharacterized protein	ISCU	3.86	.032	Posttranslational modification
A0A287ADU8	Uncharacterized protein	CLPX	2.17	.000	Posttranslational modification
D3K5K2	Peptidylprolyl isomerase	FKBP4	2.16	.010	Posttranslational modification
A0A287A3E7	Transferrin receptor protein 1	TFRC	2.09	.031	Posttranslational modification
F1SQS3	CTP synthase	CTPS2	2.78	.005	Nucleotide transport and metabolism
F1SF78	CTP synthase	CTPS1	2.63	.001	Nucleotide transport and metabolism
I3L712	Uncharacterized protein	PFAS	2.12	.028	Nucleotide transport and metabolism
A0A1L1YNR3	Fatty acid synthase (fragment)	FASN	4.19	.002	Lipid transport and metabolism
B8XSJ0	Acylglycerol kinase	AGK	3.05	.012	Lipid transport and metabolism
A0A287B6T6	Uncharacterized protein	MVK	4.17	.032	Lipid transport and metabolism
A5Z221	Acetyl‐CoA carboxylase alpha	ACACA	3.51	.015	Lipid transport and metabolism
F1SMG8	3‐hydroxy‐3‐methylglutaryl coenzyme A synthase	HMGCS1	3.05	.002	Lipid transport and metabolism
Q6RWA7	Acyl‐CoA desaturase	SCD	2.67	.020	Lipid transport and metabolism
A0A286ZLY2	Uncharacterized protein	LCLAT1	2.34	.003	Lipid transport and metabolism
A0A287AB49	Diphosphomevalonate decarboxylase	MVD	2.11	.016	Lipid transport and metabolism
A0A287APD5	Uncharacterized protein	ACSL3	2.10	.005	Lipid transport and metabolism
A0A287AN14	Uncharacterized protein	PPP3CC	5.56	.012	Inorganic ion transport and metabolism
F1S5U9	Calcium‐transporting ATPase	ATP2C2	4.47	.028	Inorganic ion transport and metabolism
M3VH94	3‐phosphoadenosine 5‐phosphosulfate synthase 1	PAPSS1	2.28	.033	Inorganic ion transport and metabolism
F1S5A6	Uncharacterized protein	SLC34A2	2.04	.008	Inorganic ion transport and metabolism
F1S451	Uncharacterized protein	LDHD	5.49	.025	Energy production and conversion
A0A287AKR7	Uncharacterized protein	HSPA14	5.20	.035	Energy production and conversion
F1RM08	Amino acid transporter	SLC1A5	2.77	.009	Energy production and conversion
A0A076JA52	NADH‐ubiquinone oxidoreductase chain 1	ND1	2.24	.013	Energy production and conversion
A0EPJ2	NADH‐ubiquinone oxidoreductase chain 3	ND3	2.13	.008	Energy production and conversion
F1S0N2	ATP‐citrate synthase	ACLY	2.10	.049	Energy production and conversion
F1SAI6	Polypeptide *N*‐acetylgalactosaminyltransferase	GALNT1	4.65	.004	Carbohydrate metabolism
A0A287ALD8	Aquaporin−3	AQP3	2.08	.021	Carbohydrate metabolism
D0G0C6	Asparagine synthetase	ASNS	2.54	.047	Amino acid metabolism
F1SUE4	Asporin	ASPN	−3.29	.046	Transcription
F1S6B5	Fibromodulin	FMOD	−2.34	.034	Transcription
A0A286ZNU3	Uncharacterized protein	CTSV	−4.12	.037	Posttranslational modification
B1Q039	CRYAB	CRYAB	−2.92	.031	Posttranslational modification
B4F449	Complement factor D preproprotein (Fragment)	CFD	−2.60	.025	Posttranslational modification
A0A287ARR1	Ferritin	FTH1	−3.44	.024	Inorganic ion transport and metabolism
F1RIP3	Ferritin	FTL	−2.61	.000	Inorganic ion transport and metabolism
I3LUD1	Superoxide dismutase	SOD3	−2.36	.022	Inorganic ion transport and metabolism
Q8WNR3	Arylsulfatase A	AS‐A	−2.11	.034	Inorganic ion transport and metabolism
F1RUM0	Uncharacterized protein	ITIH5	−2.28	.001	General function prediction only
K7GKY0	Uncharacterized protein	CD109	−2.08	.028	General function prediction only
A0A286ZLP5	Uncharacterized protein	METTL9	−3.52	.022	Coenzyme transport and metabolism
A0A286ZV82	Uncharacterized protein		−3.01	.029	Coenzyme transport and metabolism
F1RZM4	Uncharacterized protein	LAMA4	−2.14	.026	Cell motility
F1SEN1	Annexin	ANXA8	−6.48	.023	Cell motility
F1SS26	Uncharacterized protein	THBS1	−3.14	.046	Cell cycle control
D0G7F7	Tropomyosin 4	TPM4	−3.07	.000	Cell cycle control
A0A287AWV5	Uncharacterized protein	LAMB2	−2.07	.021	Cell cycle control

### 3.4 Effects of dietary valine supplementation on the expression of FASN and ACACA proteins in mammary tissue

**FIGURE 4:**
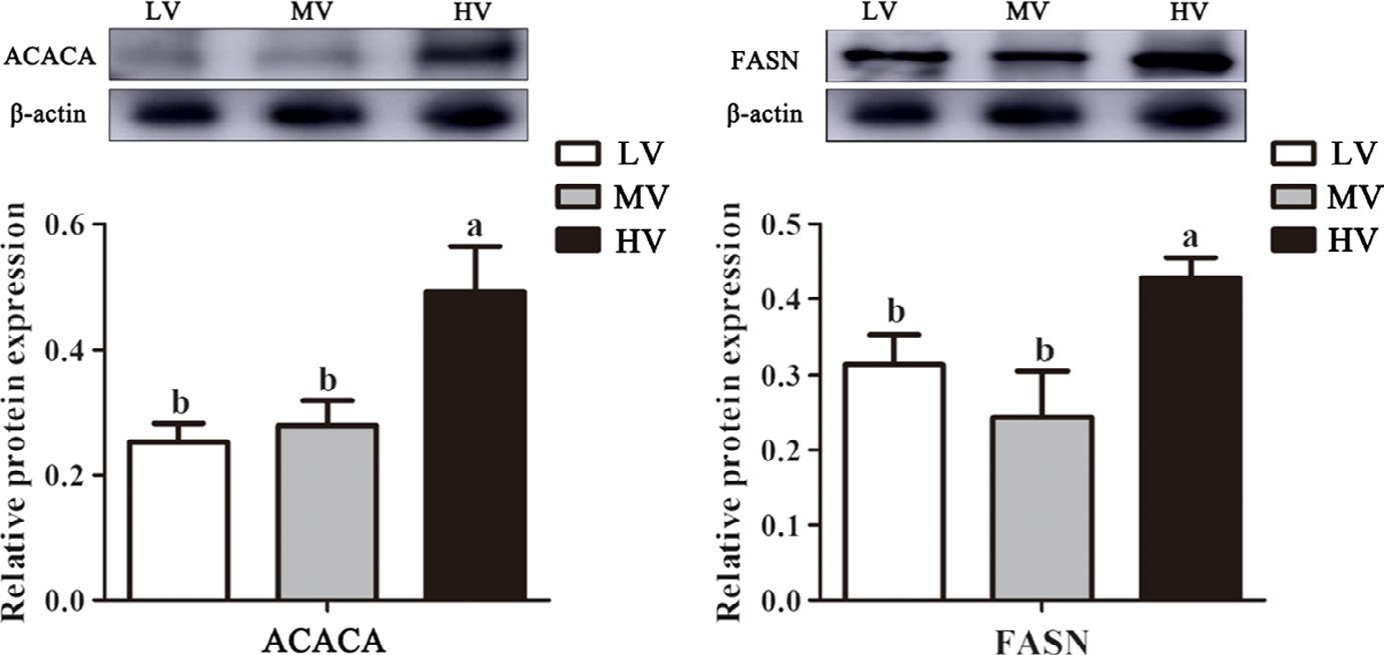
Western blotting validation of differentially expressed proteins in mammary tissue between the HV and LV groups. Note: LV: total valine:lysine = 0.63:1; MV: total valine:lysine = 0.73:1; HV: total valine:lysine = 0.93:1. FASN: fatty acid synthase; ACACA: acetyl‐CoA carboxylase. All data with error bars represent mean ± *SEM*. Means not sharing the same letter are different (p < .05)

FASN and ACACA are two important rate‐limiting enzymes involved in de novo FA synthesis in mammary gland cells. Therefore, we investigated the protein expression levels of FASN and ACACA in mammary tissues using Western blotting. The results showed that the expression of FASN and ACACA was significantly increased (p < .05) in the mammary tissues of gilts from the HV group when compared with the MV and LV groups (Figure 4).

## 4 DISCUSSION

BCAAs have a variety of physiological and metabolic functions, including glucose metabolism, cell growth, and intestinal barrier function (Doi et al., 2003; Li et al., 2009; Zhang et al., 2018). The present study demonstrated that dietary valine supplementation regulates FA metabolism and increases the milk lipid content in the colostrum, using a pig model. Our previous research showed that the colostral fat content was significantly increased with increasing dietary valine allowance (total valine:lysine of 0.87 versus 0.57) (Che, Xu, Gao, Wang, et al., 2019. However, the NRC (2012) recommended a total dietary valine:lysine of 0.73:1 during late gestation of gilts; however, we were still unsure whether it was necessary to increase valine supplementation in our earlier study. Our current study suggested that a total dietary valine:lysine of 0.93 was optimal to achieve a higher milk fat content in the colostrum than the total dietary valine:lysine of 0.73. These results highlight the relationship between valine and FA metabolism in the mammary gland. It has been reported that milk FAs in gilts mainly comprise long‐chain FAs (16–22 carbon atoms) and medium‐chain FAs (8–14 carbon atoms) (Barber et al., 1997). The long‐chain FAs in milk are mainly derived from dietary fat and mobilization of body fat (Neville & Picciano, 1997), whereas medium‐chain FAs and a proportion of FAs with 16 carbon atoms mainly come from de novo lipogenesis (Dils, 1986). In this study, gilts in the HV group had an increased content of C14:0 and C16:0 when compared with the MV and LV groups, which may be due to an increase in de novo FA synthesis with valine supplementation. As there were slight differences in the loss of back fat thickness between the three treatment groups (Che et al., 2020), it is unlikely that this phenomenon would have an influence on the milk lipid content. Moreover, all gilts were provided with the same dietary ingredients and nutrient content, except for valine. Therefore, the increased milk fat content is likely due to an increase in de novo FA synthesis. Similarly, a recent study reported a surprising finding that sows fed with a diet supplemented with glutamine and glutamate had an increased fat content in the colostrum (+60%) on Day 1 of lactation and in mature milk (+33%) on Day 21 of lactation (Aquino et al., 2014). A previous study also demonstrated that increasing dietary valine significantly increased the milk fat content (linear, p < .01) (Richert et al., 1997). Unfortunately, the role of valine in regulating de novo FA synthesis remains unclear. We speculated that valine activates the key signaling pathway or promotes de novo lipogenesis by acting as a substrate. A previous in vitro study by our group demonstrated that valine enhanced de novo FA synthesis by upregulating the protein expression of ACACA and FASN through the activation of the Akt/mTOR/SREBP1 signaling pathway in porcine mammary gland epithelial cells (Che, Xu, Gao, Wang, et al., 2019. In addition, beta‐hydroxyisobutyrate is a metabolite of valine which may be an ideal substrate for de novo lipogenesis (Mardinoglu et al., 2017). Therefore, further research is necessary to determine if valine is used as a substrate for de novo lipogenesis.

In order to further understand the mechanism of valine‐dependent regulation of FA synthesis in the mammary gland, we carried out an experiment in an in vivo animal study and investigated the related factors using mammary tissue proteome analyses with DIA. Following further analysis using proteomics, we found that 68 proteins were upregulated and 53 were downregulated in the HV group when compared with the LV group. Among these upregulated proteins, 29 were categorized according to the COG function classification, including posttranslational modifications, energy production and conversion, and nutrient transport and metabolism. Strikingly, 9 upregulated proteins, including acyl‐CoA desaturase, diphosphomevalonate decarboxylase, HMGS, FASN, AGK, and ACACA, were involved in lipid transport and metabolism. HMGS is predominantly expressed in the liver and is an important rate‐limiting enzyme involved in cholesterol synthesis (Rodwell et al., 1976). AGK is one of the most important multi‐substrate lipid kinases, which catalyze the production of lysophosphatidic acid and phosphatidic acid from monoacylglycerol and diacylglycerol, respectively (Epand et al., 2007; Waggoner et al., 2004). Furthermore, lysophosphatidic acid is considered a product of the lipid synthesis pathway and has growth factor–like activities that regulate target cells by activating a specific G protein–coupled receptor (Jalink et al., 1994; Moolenaar et al., 1997). Therefore, the increased expression of HMGS and AGK in the mammary gland is likely indicative of the enhanced synthesis of cholesterol and lipids. Notably, FASN and ACACA have been established as enzymes that play a crucial role in de novo FA synthesis in mammary gland cells, and their mRNA expression is strongly associated with de novo lipogenesis (Bauman et al., 2011). In this study, we found that the expression of the proteins FASN and ACACA in the HV group was 4.19 and 3.51 times, respectively, higher than that in the LV group, suggesting that de novo FA synthesis may be enhanced by dietary valine supplementation. Furthermore, a deeper investigation of all these proteins, performed using the KEGG pathway database, revealed that the FA biosynthesis pathway (including the proteins FASN and ACACA) was the most activated pathway. In addition, after KEGG enrichment analysis, we found that FA metabolism and the AMPK signaling pathway were significantly enriched, including FASN and ACACA. These findings have interesting implications for the regulatory function of the AMPK signaling pathway in FA synthesis in the mammary tissue of gilts. AMPK is an energy sensor that maintains cellular energy homeostasis, and previous studies have shown that AMPK activation can reduce FA synthesis by inhibiting the mTOR/SREBP1 signaling pathway and downregulating ACC and FASN expression (Quan et al., 2013; Yang et al., 2009). These findings suggest that valine downregulates the protein expression of AMPK because the expression of ACC and FASN was increased in our experiment. Similarly, Wilson et al. (2011) revealed that leucine could decrease AMPKα phosphorylation and AMPK activity as it could provide energy by converting to ketoisocaproate and oxidization via the tricarboxylic acid cycle. In contrast, beta‐hydroxyisobutyrate is a metabolite of valine, an ideal gluconeogenic substrate; therefore, valine may serve as a potential energy source for regulating AMPK activity (Cole, 2015). Furthermore, we found that oxidative phosphorylation was significantly enriched and the differentially expressed proteins NADH: ubiquinone oxidoreductase (Complexes 1 and 3), NADH dehydrogenase 1 beta subcomplex 6, and ATP synthase protein 8 were significantly upregulated in the mammary tissue of the HV group, suggesting that energy metabolism increased with valine supplementation. Overall, these results reveal that valine plays an important role in promoting the synthesis of milk lipids in the mammary tissues of gilts.

## 5 CONCLUSIONS

Our results demonstrated that dietary valine supplementation enhanced the content of milk fat in the colostrum of gilts via increased FA biosynthesis in the mammary tissue. This study is the first to provide evidence of an alteration in the response of the proteome in the mammary tissues to different levels of dietary valine supplementation in gilts, and it also demonstrates that fatty acid synthesis–related protein levels (ACACA and FASN) are regulated by valine. These findings provide new insights into the mechanisms by which valine is involved in lipid metabolism, thereby improving our understanding of lactation.

## 6 ETHICAL STANDARDS

All experimental procedures followed the current law regarding animal protection (Ethic Approval Code: 5YXK2016‐0165) and were approved by the Guide for the Care and Use of Laboratory Animals prepared by the Animal Care and Use Committee of Guangdong Academy of Agricultural Sciences.

## 7 CONSENT FOR PUBLICATION

The authors approved the consent for publication.

## CONFLICT OF INTEREST

All authors read and approved the final manuscript. The authors declare that there are no conflicts of interest.

## AUTHOR CONTRIBUTIONS

**Long Che:** Funding acquisition (equal); Project administration (equal); Writing‐original draft (equal). **Mengmeng Xu:** Project administration (equal); Writing‐original draft (equal); Writing‐review & editing (equal). **Kaiguo Gao:** Methodology (equal); Validation (equal). **Li Wang:** Conceptualization (equal); Formal analysis (equal); Investigation (equal). **Xuefen Yang:** Methodology (equal); Visualization (equal). **Xiaolu Wen:** Data curation (equal). **Hao Xiao:** Software (equal). **Mengyun Li:** Writing‐review & editing (equal). **Zongyong Jiang:** Resources (equal); Supervision (equal).

## Supporting information

Table S1

Table S2

Table S3

## Data Availability

The data used to support the findings of this study are available from the corresponding author upon request.
